# The Evaluation of Virtual Reality Fire Extinguisher Training

**DOI:** 10.3389/fpsyg.2020.593466

**Published:** 2020-11-09

**Authors:** Mina Saghafian, Karin Laumann, Ragheeba Sadaf Akhtar, Martin Rasmussen Skogstad

**Affiliations:** Department of Psychology, Faculty of Social and Educational Sciences, Norwegian University of Science and Technology, Trondheim, Norway

**Keywords:** fire extinguisher training, virtual reality, safety, realism, convenience

## Abstract

The aim of this research was to explore trainees’ perceptions and evaluation of Virtual Reality fire extinguisher training. Virtual Reality technology is being adopted by many industries for various purposes including safety training for safety critical industries. The future direction of Virtual Reality training requires an understanding of trainees’ evaluation of it; this fact motivated this research. Data were collected from 85 participants using a questionnaire after the training. Observation notes were taken to provide a better understanding of the context. Qualitative research with a thematic analysis was used to analyze the data. The results of this analysis revealed that the most salient themes reflect on issues surrounding the realism of the Virtual Reality simulation, namely different emotional and bodily experiences during the training, while the benefits of the training (health, safety, environmental advantages, efficiency and convenience, repeatability and variety of scenarios) make it a good supplement. Nevertheless, improved realism is needed to make it more effective and enhance transfer and acceptance. This study encourages the consideration of important matters (such as realism and emotions) when using Virtual Reality for fire training. It also describes the positive perceptions of this type of training (repeatability of training, safety and environmental concerns).

## Introduction

Nowadays, an increasing number of organizations have created a Virtual Environment (VE) and implemented Immersive Visual Technologies (IVT) comprising of Virtual Reality (VR) and Augmented Reality (AR) as the imminent future. They see potentials in terms of its reduced time for training and infrastructure, reduced operational costs, reduced labor costs and increased productivity, while ensuring the safety of people and facilities. The applications of IVT have expanded massively in the entertainment industry but they are also spreading to safety critical industries. One of the new trends is the use of VR for safety training in such industries.

The aim of this research was to evaluate the use of VR in safety training. Indeed, this study took an explorative approach toward the use of VR in fire extinguisher training courses aimed at safety critical industries. This is a rather new approach to training in this field and it is vital to comprehensively explore and understand the relevant issues that may have an influence on the evaluation, acceptance and effectiveness of this type of training. Furthermore, as the participants are from different organizations, their input is quite valuable as a result of their diversity and reflections on what the general working public thinks of VR trainings for safety purposes and how we can use this knowledge to improve training designs, as well as the effectiveness and transference of learning from VE to the real environment.

The host organization is ‘RelyOn Nutec’ and is based in Trondheim, Norway. They recently started to offer VR fire extinguisher training as part of broader safety training for the Norwegian oil and gas sector. This VR training application is developed by ‘Real Training,’ an organization based in Oslo, Norway. The original idea was to develop a basic fire extinguisher training for non-professionals such that one acquires and rehearses the firefighting skills in an environment similar to users’ working environment reflected in simulation. The initial assumptions were that the user must use extinguisher or water hose as in real life, to operate the fire capsule in the same way as in the real life and the capsule must have the same weight and the fire must develop, grow, respond to extinguisher and produce smoke in the same way as in real life. They collaborated with experts on fire development and created a program that simulates fires and extinguishing effects. This software is placed in a virtual reality setting and improved during an iterative process in collaboration with customers.

The increased knowledge could help all the stakeholders involved become more aware of how the training is perceived and what themes are important to consider. This will further help to make informed decisions concerning the next steps in the use of IVT in safety related trainings and further improve and adjust to the required levels of safety training. In the next section, the main theoretical background and related work are presented.

### Immersive Visual Technology

Virtual reality is a form of IVT that is defined as a simulated interactive environment in which the computer can sense and track the position and movement of the participants and “replace or augment the feedback to one or more senses, giving the feeling of being mentally immersed or present in the simulation” (Sherman and Craig, 2002, p. 9). Its features include interaction, immersion and imagination where the human perception is also involved (Burdea and Coiffet, 2003). In this paper, we focus on VR, which is “a flexible tool for investigating a wide-range of human behaviors in high-fidelity with perfectly replicable conditions” (Weech et al., 2019, p. 2). The major obstacles facing VR expansion is the generation of an ultimate sense of presence; the second barrier is the possible experience of simulator sickness, defined by Weech et al. (2019) as bodily discomfort when exposed to VR such as the experience of motion sickness. The constructs that are most commonly associated with VR environment and experience are presented next.

### Realism, Fidelity and Immersivity

The use of terminology regarding simulated environments has not been particularly clear in the literature; terms such as realism, fidelity and immersions are closely related. Consequently, there is no clear consensus on what they mean and how they differ.

In VR, the term realism is difficult to define, as indicated by Chalmers and Ferko (2008, p. 19) that “no-one can precisely define what realism is.” As a result, it is easier to refer to the level of realism, which is about the equivalent mapping of an experience in real and simulated environments. Another term used for realism is “believability,” which refers to adding as many features to the simulated environment as possible to resemble the real world (Chalmers and Ferko, 2008) and therefore make it seem or feel real.

For simulation to be realistic, the objects must seem and act in a realistic way to induce a sense of realism. This applies to when the objects are static, as well as when they are dynamic and they must be consistent in doing so (Slater et al., 2009). In order to ‘seem real,’ the virtual world must look real (visual realism), sound real (auditory realism) and feel real (haptic realism). For example, when it comes to visual realism, two components are highlighted in the literature: firstly, the extent of resemblance between virtual and real objects, known as geometric realism; secondly, the lighting model fidelity known as illumination realism (Slater et al., 2009). Realism has also been referred to as graphical fidelity (Wilcox-Netepczuk, 2013) where fidelity has been defined as “the extent to which the VE emulates the real world” (Alexander et al., 2005, p. 4). However, fidelity can expand beyond more visually oriented graphical fidelity to other elements to replicate the real world.

We believe that fidelity is about the extent of conformity to the real world, the exactness of the replication of the real world. As such, the notion of fidelity encompasses physical fidelity (to what degree the simulated environment looks, sounds and feels like the real world), functional fidelity (to what degree the simulated environment performs similarly to the real operation task and feedback) and psychological fidelity (to what degree the simulated environment produces the same psychological responses and engagement as in the real world), of which the first two overlap with realism; the latter, on the other hand, is related to the sense of presence (Alexander et al., 2005, p. 4). Fidelity in different sensory models brings about a VE that seems real to the user and engages them. This is referred to as immersion.

Immersion is about the extent to which one feels absorbed in the experience (Witmer and Singer, 1998). According to Slater (2003, p. 1), immersion involves “what the technology delivers from an objective point of view.” It is the “objective level of sensory fidelity a VR system provides” (Bowman and McMahan, 2007, p. 38) and therefore it occurs when technology can create an illusion of reality which is “inclusive (denoting the extent to which physical reality is shut out), extensive (the range of sensory modalities accommodated), surrounding (the size of the field of view) and vivid (the display resolution, richness and quality)” (Alexander et al., 2005, p. 6). According to Alexander et al. (2005), having a sense of control in the VE and smooth interaction with the VE enhances immersivity. Interaction and control require tracking potentials and functionalities embedded in the system. Slater (2003) described immersion as the extent to which the technology can deliver different sensory modalities and tracking potential that resembles the real-life sensory modalities and experience, regardless of the human perception of it. It is therefore a technology induced feature. Immersivity is enhanced if there is a higher coherence between the different sensory modalities and a higher level of realism (Cheng and Cairns, 2005), as well as realistic interaction with the VE, such as a walking interaction (Lee et al., 2017). According to Lee et al. (2017), the realistic expression of human motions accompanied by feedback according to their actions leads to presence.

### Presence

While fidelity, realism and immersion refer to the technological features of the VE that make it seem and feel real, and engage the user, presence is when the users feel like they are in the VE. Weech et al. (2019, p. 2) define presence as “the observer’s sense of psychologically leaving the real location and feeling as if transported to a virtual environment.” It is a sense of being there (Wilson, 1997). According to Bowman and McMahan (2007, p. 38), presence is a “subjective psychological response to a VR system.” Slater (2003, p. 2) states that “presence is a human reaction to immersion” and it induces the feeling of being in a real-life situation and behaving in such a way despite the cognitive awareness that it is not. While immersion is related to the system and is an objective feature, presence is related to the human in question and is a subjective experience (Gilbert, 2016) and, unlike immersion as defined by Slater (2003) being objective and measurable, presence is subjective and therefore may vary across users (Bowman and McMahan, 2007).

There have been many variables associated with the sense of presence in VE. The influential variables in creating a sense of presence can be divided into two categories: media related (including media form and media content) and user related (Riva et al., 2003). Media form variables are the physical and objective media features such as the presented sensory information, the users’ level of control over the sensory mechanisms and the users’ ability to influence the VE (Sheridan, 1992). The media content is about how both the objects in VE, and the sequence of events engage the user (Sheridan, 1992). The sense of presence for users will be different “based on differences in perceptual-motor abilities, mental states, traits, needs, preferences, experience” (Riva et al., 2003, p. 3). This notion of presence as being medial and user related, as suggested by Riva et al. (2003), depicts presence as encompassing both technology features (media form and content relating to the display technology and design), which overlaps with realism, fidelity and immersion, as well as users’ characteristics (their psychological perception and interaction with the VE) and not a psychological reaction to the system only. Consequently, the scope of what constitutes presence in the literature is not clearly distinguished.

### Multisensory Feedback

Despite the ambiguity regarding presence, previous research has shown that the sense of presence increases as more sensory modalities are simulated in the VE (Gallace et al., 2011). Different sensory modalities such as visual, audio, haptic sensory modalities have been integrated into the VE. Haptic has been more challenging to synthesize but it was attempted using either gloves or mechanical limbs (Blascovich et al., 2002). The haptic and olfactory simulation of heat and smell of smoke is less explored in the literature (Shaw et al., 2019). Nevertheless, past research has shown that the addition of thermal haptic feedback was not found to influence performance significantly, but it did improve the sense of presence and satisfaction in the trainees such that they experience lower simulation sickness, higher realism and engagement (Barbosa et al., 2017). Thermal haptic feedback was an important addition in multisensory feedback for improving realism and sense of presence (Nam et al., 2005). Incorporating haptic feedback, such as vibration, into emergency evacuation trainings was found to be effective, meaning that trainees made fewer error and finished the evacuation drill at higher speed (Jiang et al., 2005). Adding haptic feedback in terms of wearing heated suits was found to influence the trainees in taking the training more seriously (Rüppel and Schatz, 2011). Nevertheless, thermal haptic feedback must be further improved, and heating must be better synchronized in order to enhance realism and presence (Garcia-Valle et al., 2017). Addition of sense of smell to satisfy olfactory simulation was also suggested for increased realism (Shaw et al., 2019).

More progress is still needed to provide a well synchronized and overall body experience when it comes to haptic-thermal feedback, if we aim for an optimum user experience and sense of realism (Shaw et al., 2019). According to Chalmers et al. (2009) in order to achieve multisensory realism, it is not necessary to have high fidelity multisensory feedback and interaction for all the human senses because humans are not attending to all senses equally at every moment. It is possible to only focus on providing high fidelity multisensory parts that the trainees pay more attention to or utilize more, for that particular task at hand. The threshold for perception and the required fidelity level can be computed and adjusted to help achieve multisensory realism (Chalmers et al., 2009). Furthermore, when it comes to the usefulness of the VR system, Wilson (1997) states that the salience and the meaningfulness of the VE is more important than how realistic it is. Wilson (1997) suggests that the level of complexity incorporated into the VE to make it seem realistic should be proportional to the task requirements as there is a trade-off between complexity and the functionality of the system.

In sum, despite the lack of clarity in how these concepts are related, our understanding and inference about these concepts is such that fidelity in different sensory modalities leads to realism. In multisensory VE, multisensory fidelity enhances realism across all senses. This in turn enhances immersivity, which is technologically induced. This enhances presence. The emotional arousal such as feeling of stress and fear and the enjoyable experience caused by gaming elements, enhance presence and feeling engaged.

Another concept that one needs to be aware of when it comes to usability of VR, is the potential experience of simulator sickness by some individuals when using VR, which can limit the use of VR. This concept is presented next.

### Simulator Sickness

Exposure to VE and the use of VR could cause certain side effects and symptoms in some users (Wilson, 1997), referred to as the experience of simulator sickness. The symptoms include feeling disorientated, headache, eye strain, paleness, nausea, dizziness, vomiting, vertigo (where one thinks that their surroundings are swirling), sweating, dry mouth and ataxia (a lack of coordination also known as postural disequilibrium; LaViola, 2000). Simulator sickness is a more general term and includes a “visually induced motion sickness (VIMS), virtual simulation sickness, virtual reality-induced symptoms and effects” (Rebenitsch and Owen, 2016, P. 101).

According to Rebenitsch and Owen (2016), simulator sickness is concerning not only because it causes discomfort but also because of its potential safety hazards if it influences an individual who is performing a hazardous task in a safety critical industry. Furthermore, the effects of simulator sickness can last for hours or even days (LaViola, 2000). Despite the awareness of such side effects associated with the use of IVT, “there is no official standard regarding the safety of such systems” (Rebenitsch and Owen, 2016, p. 102).

Researchers have tried to understand why simulator sickness occurs. The most widely accepted theory posits that it is due to conflict between the vestibular system and the visual system, known as the ‘sensory conflict theory’ (Reason and Brand, 1975); however, there is no single theory that can explain it fully, predict it and account for individual differences in the experience of simulator sickness (LaViola, 2000). A number of contributing factors to simulator sickness have been identified that can be grouped into two categories, technology- related and individual-related factors. Technology-related factors include the quality of the visual displays, the quality of the position trackers, frame update rate and system time lag in providing real time experience and perceiving flickers which may increase as the Field of View (FOV) expands.

Furthermore, current technologies attempt to increase the FOV in VE. According to Lin et al. (2002), a wider FOV results in heightened spatial awareness, increases immersion and elevates the sense of presence. It may enhance memory if the users have a feeling of being in the VE rather than merely observing something. However, it also increases the possibility of experiencing simulator sickness. Therefore, there may be a trade-off. Females are considered to suffer more from simulator sickness, but recent studies argue that it may be due to the interpupillary distance fit of the HMDs that is less tailored to females rather than gender differences (Stanney et al., 2020).

Several studies have investigated the relationship between individual factors such as gender (see Biocca, 1992; Schuemie et al., 2005; Munafo et al., 2017), age (see Biocca, 1992; Pausch et al., 1992; Johnson, 2005), personality (Biocca, 1992; Golding, 2006), experience with VR (Uliano et al., 1986) and experience of simulator sickness. These studies have identified mixed results, meaning that the individual-related factors are not fully understood. According to Frank et al. (1984), being ill, having an ear infection, having influenza, being sleep deprived, hungover, stressed, or experiencing fatigue could increase one’s susceptibility to simulator sickness.

When it comes to the interaction between the user and the VR, sickness symptoms were found to be lower when the individual could control the objects in the VE rather than merely observing them (Chen et al., 2011). Lee et al. (2017) proposed that simulator sickness can be reduced when the individual can walk in the VE and there are walking interactions while the sense of presence is increased.

Another aspect in VR training usability is its effectiveness. As this paper is about VR training, it is important to first present what makes a training effective. This topic is discussed next.

### Training

The concept of training revolves around “planned and systematic activities designed to promote the acquisition of knowledge (i.e., need to know), skills (i.e., need to do), and attitudes (i.e., need to feel)” (Salas et al., 2012, p. 77). The desired outcome is cognitive change (enhanced knowledge), behavioral change (new or better skill level) and affective change (enhanced motivation and sense of efficacy) (Kraiger et al., 1993; Salas et al., 2012).

The effectiveness of training, referred to as the learning transfer, is the degree to which the material learned during real-life training or simulator training affects job performance (Salas et al., 2012). Such learnings should be transferrable from a structured training environment to the unstructured and unpredictable real-life context (Alexander et al., 2005). A positive transfer refers to situations in which the training leads to improved performance in an applied setting. However, if the training compromises or lowers the quality of one’s performance in the applied setting, negative training has taken place (Alexander et al., 2005), possibly due to the incoherence between actions and behaviors learned in the training and needed in the job. Other issues that might have a negative effect on training quality could be intense presentation of materials without involving the trainees, distraction by other trainees and skill degradation (Tichon and Burgess-Limerick, 2011). An effective way for safety training is the use of serious games. This is further explained next.

### Serious Games

Virtual reality technology is becoming very popular for training for world situations through using serious games (Chalmers et al., 2009). The term serious games refers to games that are not merely for entertainment but also for educating people about situations (Feng et al., 2018) that are hard to replicate in real world due to safety concerns and lack of time and resources (Susi and Johannesson, 2007). In addition to being educational (Backlund et al., 2007a; Susi and Johannesson, 2007), serious games are also dynamic (Chalmers et al., 2009) and provide contextual experience and interaction with the virtual environment, teaching trainees how to strategize and use their knowledge and skills (Oliva et al., 2019). Serious games include a success or failure outcome and provide instant feedback on performance (Oliva et al., 2019).

Prior work has shown that serious games is a feasible method to train fire fighters (Backlund et al., 2007a) and offers a better training for learning safety instructions compared to the traditional safety cards, because it entices feeling of fear and enhances engagement (Chittaro and Buttussi, 2015). VR serious game offers the advantage of providing individual feedback and more emotional engagement (Backlund et al., 2007a) compared to traditional training. This impacts trainees’ emotional and physiological arousal, (Chittaro and Buttussi, 2015), attitude and behavioral modification in emergency situations (Feng et al., 2018) and acquisition of new skills (Backlund et al., 2007a). In the serious game VR training for fire evacuation, trainees indicated being highly engaged and found the platform very appealing (Oliva et al., 2019). Furthermore, since it does not interrupt the organizational routines and work flow while training, employers are more likely to embrace this type of trainings compared to real life drills (Chittaro and Ranon, 2009). The learning effect of VR serious games was mentioned to be further enhanced if it was supplemented with other modes of training, more frequent training and more teamwork during training (Wouters et al., 2013).

### Related Work on VR Safety Training Domains and Effectiveness

When it comes to safety, training is especially important to prepare trainees for procedures that need to be followed in emergency and disaster situations where there is a high level of stress and negative emotional arousal. However, traditional training methods in safety critical industries can be costly and impractical, if not impossible (Andreatta et al., 2010). Access to repetitive and on-demand training is limited, context is inconsistent and individual feedback is suboptimal (Andreatta et al., 2010).

VR training could address these limitations and be used for simulation and training (Melo et al., 2016). It is a suitable and portable solution for continuous training in various fields and industries (Al-Adawi and Luimula, 2019). Previous work has shown a wide range of areas where VR safety trainings have been applied. Kinateder et al. (2014), conducted a review of how VR has been applied for human behavior studies in various disaster and emergency situations including fire-related trainings (Cha et al., 2012; Xu et al., 2014; Farra et al., 2015) and evacuation training (Kobes et al., 2010; Rüppel and Schatz, 2011; Bode et al., 2014; Kinateder et al., 2014). In a review done by Feng et al. (2018), on the application of VR serious games for disaster related training, it was shown that the majority of studies have been done, in turn, on fire evacuation, aviation, spacecraft and earthquakes (Feng et al., 2018). Tate et al. (1997) studied the use of VR for shipboard firefighting and its effectiveness in both developing skills and navigation techniques using simulated smoke and fire (Tate et al., 1997). Navigation techniques were found to be influenced through formation of cognitive map and gaining spatial knowledge using VR simulation (Cao et al., 2019).

Chittaro and Buttussi (2015), have also provided an overview of safety related studies using VR, including fire safety and evacuation (Backlund et al., 2007a; Mól et al., 2008; Cha et al., 2012; Bachen et al., 2016). In addition to that, they also presented another examples of how VR training could be used for safety in other areas, such as traffic safety (Backlund et al., 2007b), risk recognition (Jorge et al., 2013), and pedestrian safety (Schwebel et al., 2008). Andreatta et al. (2010), have studied the use of VR for training in medical emergency and dealing with mass-casualties (Andreatta et al., 2010). VR training has also been applied for promoting safety in construction industry (Gao et al., 2017, 2019). This shows the various domain in which VR can be used for training.

As for the effectiveness of VR training, it was posited to be an effective alternative to conventional training methods for navigation (Bliss et al., 1997). Chittaro and Ranon (2009) found that serious game VR was more effective in acquiring safety knowledge and in memory retention, in comparison to conventional methods such as reading safety cards. VR training for firefighting skills for inexperienced trainees was reported to be effective in learning fundamental skills of firefighting (Cha et al., 2012). VR training was also found to be effective for safety training in construction, in comparison to training with other computer aided technologies (Gao et al., 2019), where effectiveness was reflected in gaining knowledge, changing unsafe behavior and lowering error and injury (Gao et al., 2019).

In the recent literature on the use of VR for fire extinguishing training, the advantage of this method compared to less interactive methods has been found. “VR training provided a more effective training result in terms of knowledge acquisition and retention, and self-efficacy” (Lovreglio et al., 2020, p. 12) in comparison with traditional video training. It was also suggested that VR training increased motivation and perception of threat compared to video training. Månsson (2018) found that trainees that did a VR fire extinguisher training in an emergency room scenario, followed by using the real fire extinguisher performed better than those who started training with real fire extinguisher. He further suggested that VR training could be a complement to the traditional methods of fire training.

In addition to the results of VR training and its effectiveness, it was mentioned that the strength of VR is in creating a controlled, immersive and safe set up (Kinateder et al., 2014). Its entertaining property motivates the trainees in using this tool (Backlund et al., 2007a) and benefit from a convenient and safe training in real time, which increases interactivity and presence, and allows for evaluating the training effectiveness (Cha et al., 2012).

Enticing emotional arousal, such as simulating stressful environment in fire evacuation training, without any physical harm is another advantage of using VR training (Zou et al., 2017; Cao et al., 2019). This emotional arousal, especially negative emotion such as fear, was found to increase memory retention which make VR fire training more effective (Chittaro and Buttussi, 2015). Acclimatization to stress through VR induced fear arousing scenarios (Tate et al., 1997), can be used to overcome cognitive paralysis as in fatal inaction during emergency situations (Chittaro and Ranon, 2009).

In order to improve the quality of training outcome, it was suggested to have more consideration of the target group and their skill level when designing the VR training. For novice trainees, a less realistic set up will suffice to train for the fundamental skills but as expertise and experience increases, realism must increase accordingly (Garcia-Valle et al., 2017). Prior experience with computers, especially gaming, was another factor that was suggested to be considered (Walkowiak et al., 2015). To improve team effectiveness in fire emergency, it was suggested to have a multi-player VR environment where trainees are able to communicate together (Cha et al., 2012). With regard to usability, it was reported that postural instability during the VR training can influence trainees’ performance (Melo et al., 2016). This highlights the importance of conducting usability analyses for human-technology interactive system to ensure that usability can be applied and customized to that particular VE and the training goal. In order to ensure usability, the VR training system must be easy to use, the task must be easy to learn and easy to remember, the training must be efficient, safe and fun for the trainee (Brinck et al., 2002; Wickens et al., 2004; Calp et al., 2012). While past studies have focused on the technology aspect of immersive systems and simulations (Vicente, 2003; Fernando Capretz, 2014), more work should be done on the human side, the trainees’ well being and satisfaction (Gao et al., 2019). This encompasses factors that affect how trainees interact with and perceive the system (Walkowiak et al., 2015) and its usability (Feng et al., 2018).

Alexander et al. (2005) argue that increasing the level of fidelity, immersion, presence, and users’ acceptance and belief in training usefulness enhance positive transfer in a simulated environment. According to Alexander et al. (2005), user acceptance creates a training mindset in which one is willing to practice the behaviors and actions promoted in the training. This highlights the importance of understanding how trainees evaluate the training and its potential impact on training outcome.

According to Salas et al. (2012), although simulators try to resemble realistic training, they are not an exact replication of the task environment. They should be “working representations” and there is no need to be an exact copy of the real world. They argue that physical fidelity is not as essential as psychological fidelity, reflected in the relevance of the content that is being offered by the simulation to the job. What matters is the design of the simulation and scenarios, and the provision of instruction, measure of performance and timely feedback (Salas et al., 2012) that trainees can learn from.

In this research we aim to explore usability, in the sense of trainees’ attitude and perspective, in order to see how they evaluate the VR training. Their insight can provide valuable input into how training can be further tailored and improved. The attitude of the trainees plays an important part in the acceptance and effectiveness of the system (Vicente, 2003). The trainees in this research are from diverse workplaces and they have different experience levels with safety training. They are all employed within safety critical industries in Norway, therefore, they are aware of importance of safety training according to the national requirements. They are all taking part in this VR training for the first time. Follow up refreshment trainings can also be conducted within VE in the upcoming years. We believe that early stage subjective evaluation can provide insightful feedback from trainees about what can be improved in the training, how can realism and training effectiveness be enhanced to further increase usability. In short, we explore how training is perceived by trainees and what can contribute to its acceptance, evaluation and effectiveness.

## Materials and Methods

In this section, the context of the training, data collection and analysis are presented. The context includes a description of the training setup, including the equipment used and the space in which the training takes place, as well as the description of the training session to provide a better overview of the training context.

### Training Setup

For the purpose of this VR serious game training, SteamVR system was used to track and process the interaction between the trainees and the VR fire extinguisher. This system included the Head Mounted Display (HMD), the desktop that runs the VR software, the two laser emitting base stations and the fire extinguisher that is equipped with sensors, the HTC Vive Pro HMD was connected to a desktop through a hard wire of approximately five meters in length. The position of the trainee was tracked by the use of two laser emitting bases that were placed in a corner of the room, close to the desktop. The laser when contacting with the sensors on the HMD, could track its position. Furthermore, there were sensors on the fire extinguisher. The trackers captured the user’s interaction with the extinguisher capsule through button mechanism routed through Vive Tracker. The developers used custom tracker based on Steam HDK (SaS) and FireSim Vive trackers for this application. The exact details cannot be provided based on the agreement with the developers. In addition to the hardware and softwares mentioned and their communication through the use of trackers and sensors, the application also included virtual scenes in each scenario that consisted of the colored static 3D objects with realistic size and shape, the exit sign, the dynamic simulated flames and smoke, and the fire extinguisher that was controlled by the trainee in each scenario. The functional requirement included rotating in order to locate the exit sign and the source of fire, moving toward fire extinguisher, lifting it, moving and aiming the hose of the fire extinguisher to the source of flames. The feedback messages corresponding to trainees’ action would indicate success or failure in putting out the fire at the end of each scenario. The performance requirements were to gain an awareness of the scenario, the direction of the exit route, the spotting of fire, aiming at the fire through suitable body positioning, spraying fire extinguisher agent and moving around accordingly to put out the flames. The design constraints included the lack of thermal feedback from the fire and the smell from the smoke.

The fire extinguisher capsule was customized to resemble a standard capsule of approximately six kilograms filled with water. The developers have a background in firefighting and have used this weight as the standard fire extinguisher weight. It also included the discharge nozzle, discharge lever and carrying handle. There was no simulated safety pin. The software used for training was from Steam VR. The training room for the indoors training was approximately 49 m squared. The movement of the trainees was limited by the length of the wire that was attached to the HMD, which was about 5 m long.

**FIGURE 1:**
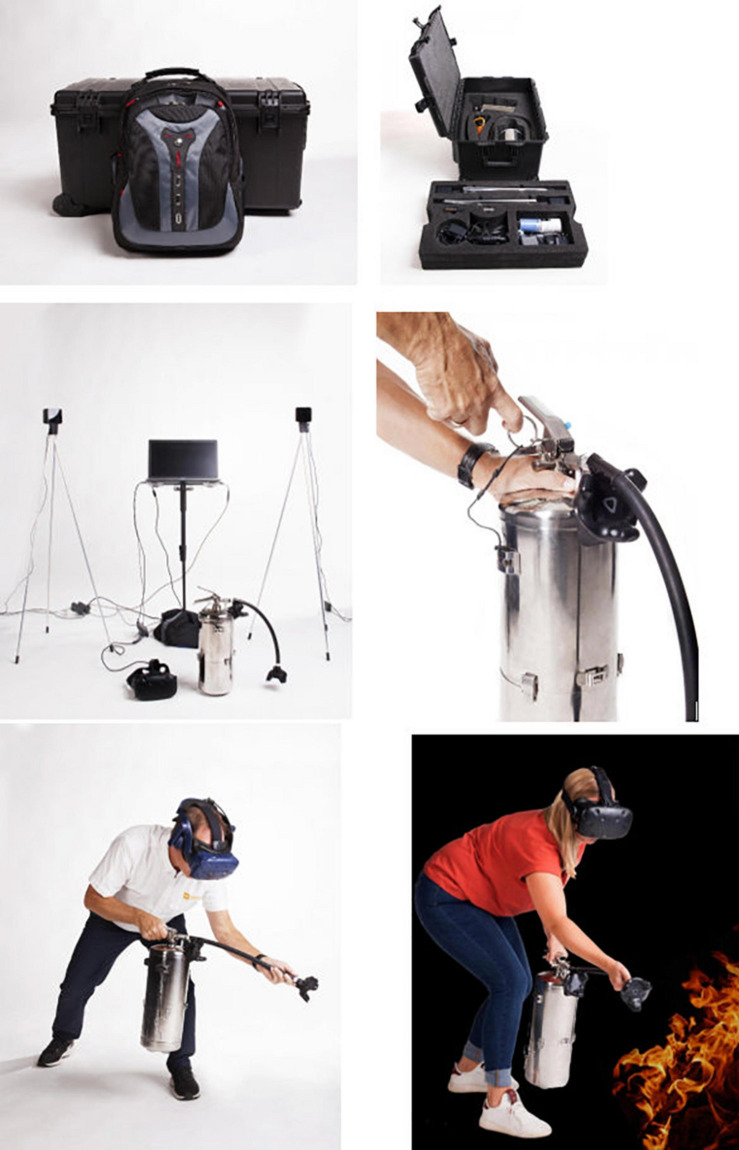
Virtual reality (VR) training application setup and use.

**FIGURE 2:**
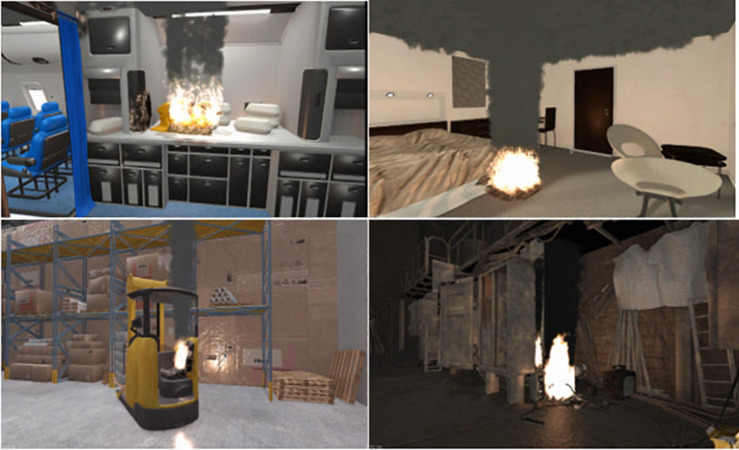
Examples of VR training scenarios.

**FIGURE 3:**
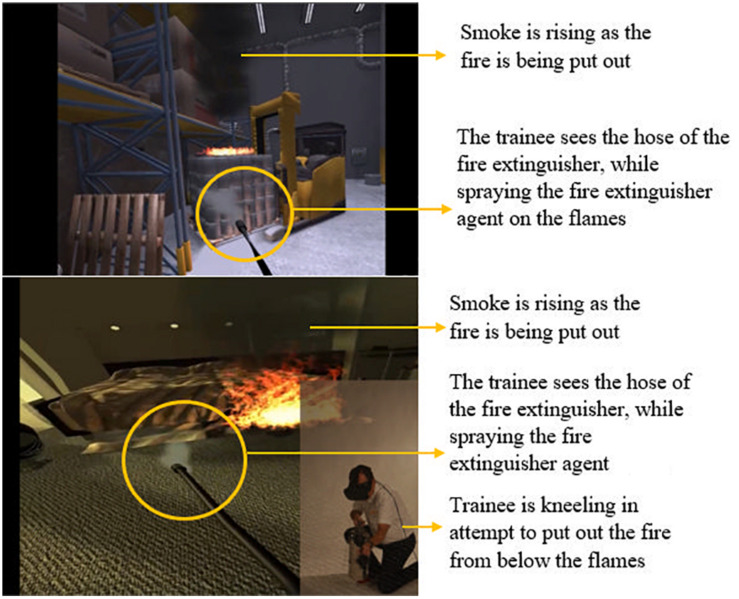
Virtual reality training scenario from the point of view of trainee when practicing fire extinguishing skills.

**FIGURE 4:**
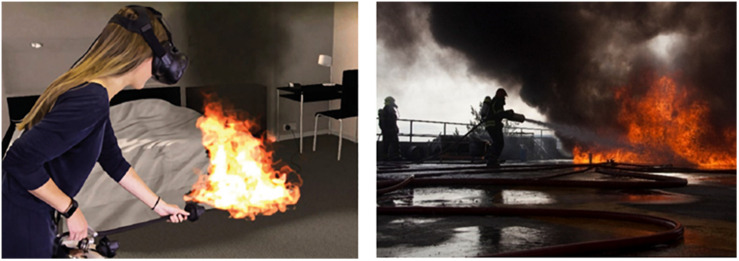
Virtual reality fire training simulations indoors **(left)** and real fire training outdoors **(right)**.

The training included six scenarios with different types of fire: airplane cabin, hotel room, control room, kitchen, warehouse and a conveyer belt in a factory, all of which were offered at ‘easy’ and ‘expert’ mode; this refers to the difficulty level such as the reaction speed needed to put out the fire. The trainers were certified by Real Training. The sessions started with a theory-based lecture about different types of fire and the use of different extinguishers, followed by the indoors VR training and then the outdoors traditional fire training. There were seven to eight trainees in each session. One trainee would be standing and moving with the fire extinguisher while the rest could watch what the person sees on the screen. The flame and smoke spread were developed in collaboration with two research institutes to resemble real flame and smoke spread. The trainer stood in a corner with a laptop to administer the scenarios while giving instructions and feedback on what to look for (where the flame is, where the exit door is, what type of extinguisher users hold in their hand), distance adjustment, posture adjustment such as kneeling to put out the fire and speed adjustment for putting out the fire. The duration of the training sessions was approximately 45 min per session but would differ depending on the number of participants and the number of questions and answers. Each trainee was able to practice all the scenarios and on average it took 6 min per person to complete the training. See Figure 1 for the VR application parts and set up, Figure 2 for examples of VR fire scenarios and Figure 3 for how the trainees would see the VR fire and engage in extinguishing it. Each trainee received feedback during each scenario by the trainer and by the cues and feedback provided within the VR training and a final ‘success’ or ‘failure’ feedback. See Figure 4 for VR fire extinguisher training in the hotel room scenario showing what the trainee sees in the VR environment taking place indoors versus the outdoors training that takes place in open space in the training facility. In the outdoors training, all the trainees had to wear protective suits and could see the effect of wind on the flames, the difficulty of putting out the fire and the need for distance adjustment. Such a large open space made it possible for trainees to stand far away from the fire. Not every single trainee got the chance to practice putting out the outdoors fire.

### Data Collection

The research project was introduced prior to theoretical training and informed consent forms were distributed and collected prior to the VR training session. When participants did not sign the informed consent forms, observation of the sessions did not take place. Otherwise, observation notes were taken during each training session. Questionnaires were handed out immediately after each training session when all the trainees present in that session were done with their training round. They filled in the questionnaire if they wished to, and left it turned on a table. The researchers left the room to avoid their presence influencing the participation in the research. They later collected the questionnaire forms to ensure anonymity and volition of participation. The questionnaire was developed by the research group and because we aimed to explore what the trainees really thought about the training and their attitude, we used open ended questions mostly. The survey was distributed after the VR training session.

### Participants

The training was performed in four rounds, each round included three groups of trainees and each group included seven to eight trainees. The questionnaire was handed out after the VR training session was finished and before the trainees left the room. A total of 85 participants filled in the questionnaire. The questionnaire used can be found in Table 1. Participants were from different organizations. Since the use of VR fire extinguisher training was introduced fairly recently, none of the trainees had experienced this particular type of VR training before. Each training session was run by one trainer. In total, two trainers were certified to teach the VR training sessions and were present on different training days. Observation notes were taken when consented to by everyone to help better understand the context and were used as background information. The observation mainly focused on the general atmosphere of the training session, the number of questions and answers, how engaged the trainees were in terms of their physical movement while trying to put out the virtual fire and their comments about the training session.

**TABLE 1:** Questionnaire that was administered immediately after the VR training session with four open-ended and two yes/no questions.

	Questions
1	Do you have previous experience with VR?
2	How do you evaluate training with the use of VR?
3	How was the VR training compared to traditional training?
4	How did you feel during the training?
5	What are the advantages and disadvantages of training with the use of VR?
6	Did you experience nausea or motion sickness during the training, and if so when?

### Data Analysis

The answers to the questions, when possible, were categorized as ‘positive’ when the comment only contained a positive viewpoint, ‘negative’ when the comment only contained a negative viewpoint, and where a comment was a mixture of positive and negative comments, it was categorized as ‘mixed.’ When a comment did not fit into any of these categories, such as ‘ok,’ it was categorized as ‘neutral.’ The results from this categorization were used to report on the percentages reflecting on the general trends in the trainees’ answers. The content of the answers and observational notes were imported into qualitative analysis software, NVivo 12 pro. A thematic analysis was conducted using the data to identify patterns or themes in the responses (Braun and Clarke, 2006) reflecting important information. This is performed by a critical reading of the responses and observation notes and an iterative coding process. This inductive coding was mainly on a semantic level.

## Results

The aim of this research was to investigate how the trainees evaluated their VR fire extinguisher training. The total number of trainees was 85, including 65 males, 10 females and 10 participants who did not disclose their gender. The average age of the participants was 40 years old. Not everyone answered all the questions. The results are presented in two parts. The first part presents the percentages of the responses to reflect on the general opinions of the trainees about the VR training. The second part presents the themes from the qualitative analysis.

### Quantitative Analysis of the Results

The quantitative overview of the results is presented in Table 2. An explanation of what can be understood from these numbers is provided next.

**TABLE 2:** A quantitative overview of the evaluation of VR for safety training.

	Response number	Yes	No	Positive	Negative	Neutral	Mixed
Previous experience	85	30	70				
VR training evaluation	83			65%	5%	8%	22%
VR versus traditional training	83			40%	19%	13%	28%
Emotional experience during VR training	82			65%	2%	29%	4%
Simulator sickness	83	10%	90%				

#### Previous Experience With VR

The questionnaire included a question about participants’ general level of previous exposure to VR. This question gives the study an idea of how familiar the trainees are with VR. The number of respondents was 82, from which the majority (70%) did not have previous experience with VR. The rest (30%) had previous experience largely through gaming, but also through their jobs, including the use of VR for geographical simulation modeling.

#### VR Training Evaluation

The responses (*n* = 83) to this question were categorized as positive (65%), mixed (22%), neutral (8%), and negative (5%), showing that most people have a positive outlook on using VR for training. The mixed responses included comments that had both a positive and a negative view about the training. The neutral comments did not reflect on their evaluation and were not sufficiently informative. An example of positive evaluation is “very good, effective, quick learning, environmental and health-promoting.” An example of a mixed evaluation is “good to get repetitions but cannot replace real training.” An example of negative evaluation is “not very real” and an example of neutral response is “Ok.”

#### VR Training Compared to the Traditional Training Evaluation

The responses (*n* = 83) to this question were categorized as positive (40%), mixed (28%), negative (19%), and neutral (13%), showing that less than half of the trainees had a positive view about the VR training compared to the traditional training, despite the fact that the majority of the trainees positively evaluated the VR training by itself.

#### Emotional Experience During the VR Training

Most responses (*n* = 82) reflected on having experienced positive feelings (65%), followed by neutral feelings (29%), mixed feelings (4%), and negative feelings (2%).

#### Experience Simulator Sickness

Most of the respondents (*n* = 83) reported not having felt any motion sickness symptoms (90%). The respondents who felt unwell (10%) reported feeling nauseous during the training, dizzy after the training and discomfort with the HMD.

### Qualitative Analysis of the Results

**FIGURE 5:**
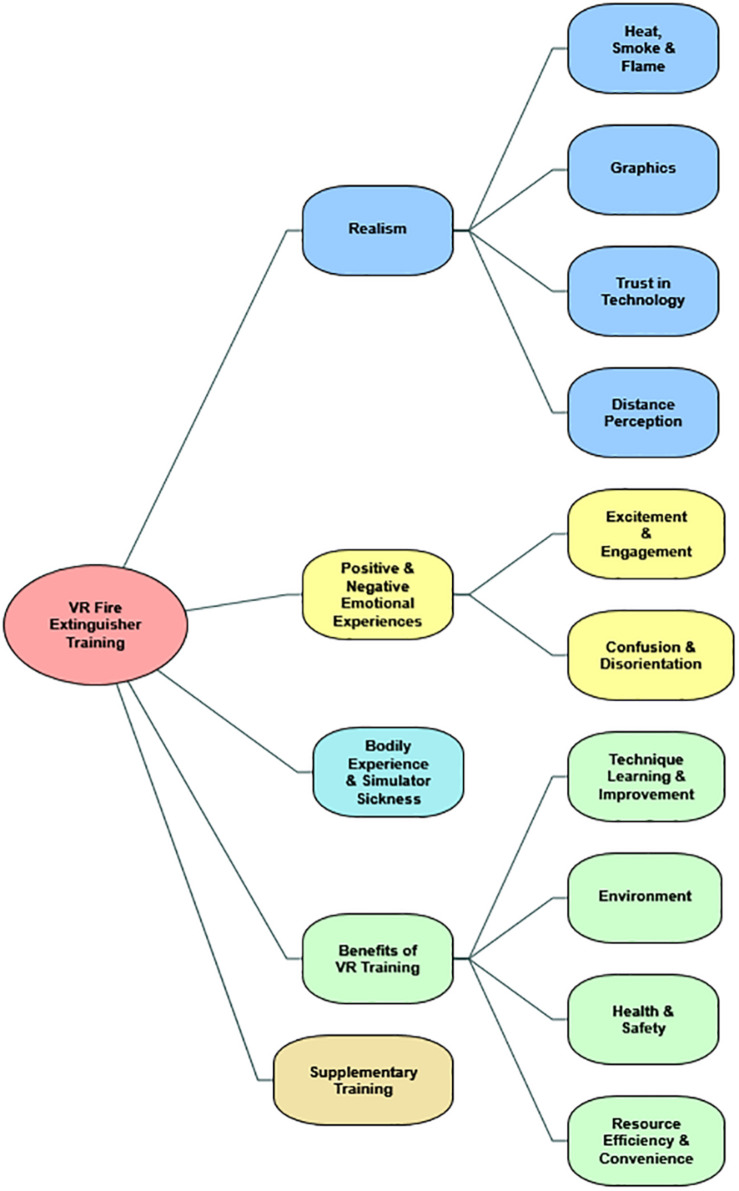
An overview of the resulting themes and subthemes of evaluation of VR training.

The second part is the qualitative overview of the themes that were derived from responses and observations with the use of the thematic analysis. An overview of the themes and subthemes are presented in Figure 5, and additional examples of quotes are presented in Table 3.

**TABLE 3:** An overview of themes, subthemes and quotes.

Theme	Subtheme	Examples of quotes
Realism	Heat, smoke and flame	(1) “A lot of stuff is good, but it’s missing the smell, warmth and how a real fire act[s]”
	Graphics	(2) “Could have had better graphics and be more realistic”
	Trust in technology	(3) “It’s difficult to trust the extinguisher’s abilities with computer generated flames”
	Technical features	(4) “Extinguisher is too light, and you don’t have to take out the safety pin” (5) “Cannot feel pressure/resistance in the tube/hose and don’t get a feeling of warmth, danger, smell and smoke”
	Distance perception	(6)“Impressed by the distance reality” (7) “Traditional training gives […] better distance assessment, it’s easier to orient oneself”
Positive and negative emotional experiences	Excitement and engagement	(8) “Very good and exciting experience” (9) “Good, became engaged”
	Confusion and disorientation	(10) “On edge and easily disoriented” (11) “A little confused, a little unusual” (12) “Ok, but got a little stressed” (13) “A little disoriented”
Bodily experience and simulator sickness		(14) “When changing scenarios experiences easily confus[ed] and nausea[ted]” (15) “Got a little nauseous and could not have done this more than 5–10 min” (16) “Dizzy/nauseous after VR” (17) “Uncomfortable with the glasses on” (18) “These are okay exercises, but can be irritating for the eyes”
Benefits of VR training	Technique learning and improvement	(19) “Looks like one can train the technique of fire extinguishing” (20) “Good for learning the method and to get many repetitions” (21) “You can train in many scenarios”
	Environment	(22) “Safe and environmentally friendly way to conduct the training with the same results” (23) “Can train more often without damaging the environment”
	Health and safety	(24) “Less damaging and better for health” (25) “Advantage when it comes to safety” (26) “Safe and good”
	Resource efficiency and convenience	(27) “Efficient, can train a lot and fast” (28) “Good that one does not need equipment to burn things” (29) “Efficient when it comes to different scenarios” (30) “Easy to use”
Supplementary training		(31) “A good supplement, but cannot substitute original training with today’s technology” (32) “Works as a supplement/additional training” (33) “I think this is the future” and “this will be successful”

#### Realism

The theme of realism is one of the most prominent themes in the VR training. It pertains to the issues that shaped the trainees’ perception of realism and what would make the training realistic in their point of view. Most comments showed that the level of realism was a concern including comments stating that the VR was “artificial” and “was not real enough,” while there were also trainees who believed that the VR experience was “just as good” and “very similar” to the real experience. This reflects on differences amongst trainees in their perceptions of realism in VR. Realism, as an overall theme, includes subthemes, namely the significance of heat, smoke and flame, the display’s graphics, having trust in technology, the technical features and the distance perception.

The lack of heat, smoke and flame was raised many times by the trainees as an issue with the VR training. They were missing the sense of warmth, smoke and smell. One of the concerns was about not getting the “live reaction” of the fire. The trainees mentioned that they missed the instant live feedback from the flame in response to their motion, distance, the technique and the extinguisher they used (see quote 1 in Table 3).

Another subtheme was the display’s graphics. One comment was that the VR display “has good graphics.” However, another comment opposed that (see quote 2) and clearly showed the trainee’s perceived link between graphics quality and the perception of realism. We believe that the opinions about the quality of graphics could be based on trainees’ prior experience with VR. It also shows that some trainees may be more aware of how technological features such as graphics can create a sense of realism due to their own experiences, while others may be less aware of this link. This could be why realism as a theme and graphics as its subtheme contained different points of view.

One drawback that was mentioned with regard to realism reflected on the lack of trust in how well a computer-generated flame actually represents real flame behavior in response to extinguishers and recreates reality (see quote 3). This can be due to a lack of previous exposure to immersive technologies such as VR, which could create a general distrust in technology, or a high level of experience with real firefighting.

Realism was also considered through the lens of technical features of the VR extinguisher compared to the real fire extinguisher and the steps that should be taken. One comment reflected on the equipment and the procedure not quite matching the reality (see quotes 4 and 5) as the trainee did not have to pull the safety pin and did not feel the pressure of the fire extinguisher agent as it was being released.

Another subtheme derived in connection to realism was distance perception. There were both positive and negative evaluations of how realistic one’s perception of their distance and positioning in VR training was compared to reality. The comments showed division on how distance was perceived, including it being realistic as well as not as good as the real fire scenario (see quotes 6 and 7).

#### Positive and Negative Emotional Experiences

The trainees describe different emotional experiences while performing the VR task that were captured under the subthemes of feeling excitement and engagement, as well as feeling confusion and disorientation. The subthemes of excitement and engagement reflected on the positive feelings reported, including “feeling good,” “feeling excited,” “becoming engaged,” feeling “comfortable” and even a progression into positive feeling as shown by the comment of “getting into [a] good mood” (see also quotes 8 and 9). There were also reports of feeling “focused,” feeling “in control” and even a report of feeling like a “superhero.”

The subtheme of confusion and disorientation reflected on negative feelings. Responses included feeling “[a] little confused” and “[a] little unusual.” There was a report of getting “[a] little stressed,” in addition to a participant mentioning “[being] on edge and easily disoriented” which could reflect a sense of unfamiliarity with the VR environment and not being used to moving in a VR setting (see quotes 10 to 13). Other responses included feeling “Ok” and “normal.” One person responded that he/she was focused in the beginning but became disappointed in the level of realism during the training. This shows a perceived link between the perception of realism and the resulting emotional experience. It is not clear if engagement and disengagement were linked to the design on the VR scenarios, the VE and its objects and how realistic they seem or the interactive fidelity experienced by users that led some to become more engaged and excited, while others became less engaged and disappointed or even stressed.

#### Bodily Experience and Simulator Sickness

This theme focuses on the bodily experiences of the trainees during the training. The minority of trainees experienced simulator sickness, including feelings of motion sickness, dizziness, vertigo, and nausea. With regard to the timing of the experience of simulator sickness, an individual difference was eminent. There were comments on only being able to bear “limited time in VR” and “getting nauseous a little during the training”, while another comment reported that switching between scenarios caused “confusion and nausea” and another trainee experienced nausea after the VR training (see quotes 14–18). This shows that the timing of the experienced side effects is different for each individual. As for other bodily discomforts, there were two comments stating that the exercises caused eye irritation and that it was “uncomfortable” to wear the glasses.

#### Benefits of VR Training

This theme includes a number of subthemes that were unanimously considered by the trainees to be advantages of the VR training. The subthemes include technique learning and improvement, environment, health and safety, resource efficiency, and convenience.

The technique learning and improvement subthemes reflected on the benefit of VR training as a supplementary educational experience and described it as “quick learning,” “effective,” and a “useful” experience. They found it to be a good way to acquire techniques, and a “good starting point.” Participants also mentioned that practicing techniques helps “to cope with stress” in the case of a real fire. Repeating the training without any costs endured and experiencing different scenarios at different difficulty levels was found to be useful and helped trainees familiarize themselves with different situations (see quotes 19–21) and improve their skills.

The environment subtheme was a prevalent subtheme mentioned as a clear advantage of the VR. Being able to train more without damaging the environment and without high carbon dioxide emissions and heat was an evident advantage (see quotes 22 and 23). This was accompanied by the subtheme of health and safety reflecting how trainees felt safe, secure and in control due to the reduced exposure and risk (see quotes 24–26) and consequently perceived it to be a safer way of training.

The subtheme of resource efficiency and convenience relates to having the advantage of repetition and the aforementioned multiple scenarios without consuming too many resources. It was noted that VR is “better than theory” for learning tactics and managing stress as one learns to “cope with stress” in VR. It requires fewer resources and is “inexpensive” and “cheaper.” Convenience of use is also related to avoidance of the heat and smoke that made the indoors VR experience easier and less stressful. The training goes faster while everyone gets the chance to experience the training (see quotes 27–30).

#### Supplementary Training

The majority of respondents believed that this training cannot replace traditional training, but it could be a beneficial supplementary training for refining skills. It still needs further development to become an alternative to real training (see quotes 31 and 32). A few trainees, however, believed it to be a “good alternative” and found it to be “quite close” or “similar” to the fire training with real fire. The reason for it being a supplement was mainly because it was not perceived to be realistic enough as it is lacking the elements of heat, smoke and flame reaction. This theme is therefore linked to the theme of realism. Nevertheless, the trainees commented on how this technology could be a successful part of the future (see quote 33), which shows an awareness of emerging industrial trends.

In the following section, the themes that were derived from the observation notes are presented. However, as they did not come from the trainees, they are merely used to better understand the context.

#### Framing of Training Process and Objectives

It was observed that the way the trainer framed the VR training and the information about it could influence the trainees’ evaluation of it. When VR training was framed as a supplement to the real training, it was perceived more positively and with potential benefits for the future. This was also the case where the trainer explained that VR can be used for certain aspects of the training, while real fire training can be used for other aspects. Furthermore, when the trainer explained about the current shortcomings of VR and also explained how they are in the process of improvement, the general perception of the VR training was more positive. There was one group that openly expressed skepticism about the use of VR as a training method and mentioned that it was too easy and not challenging enough. They did not fill in the survey. In this group, VR was not introduced as a supplement nor a work in progress. In one data collection day, consisting of three rounds of data collection in three different groups, it was observed that when the trainer started the VR training session with more challenging and difficult levels of VR scenarios, the trainees seemed more engaged in comparison to previous groups that started with easier levels. The way that the trainer introduces and implements the training sessions and what they say about the role of VR training in the safety training package as a whole also influences the attitude and evaluations of the trainees. This effect is interesting grounds for further research.

#### Group Dynamics

It was observed that in the groups where there was a more positive atmosphere and more conversation and, the trainees seemed more engaged and were actively trying to put out the fire and seemed to enjoy it. There was a higher level of openness toward this new way of training and people seemed to be more positive. It could be that the opinion of one person influences the following discussions and shapes the attitudes of the others at least for the time being. However, the observation and the survey data cannot determine the exact relationship between the group dynamic and the evaluation of the VR training. Furthermore, when the trainer made more jokes, the atmosphere became more amiable and relaxed. The group seemed to be less tense but not less engaged.

#### Trainees’ Point of View About the Future of the Training

Toward the end of the training, the trainees started discussing about how they thought VR was going to be developed in the future. While they mentioned it to be a supplement, they also saw potential for its future. They mentioned that VR technology was getting better and it will be use more and more. In general, trainees saw it as an educational tool that would get better in the future. The main issue that they mentioned was realism and that it needed to become more realistic. They also stated that it needs to become more challenging.

## Discussion and Future Implications

The use of VR for safety training was evaluated through questionnaires and observation notes. The trainees were employees in safety critical industries, but they were from different educational and expertise backgrounds and were mainly male participants. The results showed that more than half of the trainees (65%) positively evaluated the VR training in general, while less than half of the trainees (40%) evaluated the VR training positively in comparison to the traditional training. Approximately 65% of trainees experienced positive emotions during the training, while 10% of trainees experienced simulator sickness during the training.

The trainees had different opinions about how realistic the VR training was, and they showed individual differences in their emotional experience during the VR training. They were unanimous on the advantages of the VR training for health and safety, the environmental friendliness of this training, its lower resource requirement and the convenience of training. As for the current state of the VR technology, the majority believed that it is a good supplementary training method that offers more than mere theory and it is good to practice techniques in different scenarios at one’s own convenience, but it still lacks the elements that make the training as realistic as traditional fire training. They also acknowledged the future potential of VR for training.

### Experience of Realism in the Training

As mentioned in the introduction, Chalmers and Ferko (2008) posit that there is no unanimous definition of realism. Therefore, evidently there are differences in evaluating realism. If we consider realism as a similar or collective result of the fidelity in every sensory modality that the VR system offers, then it could be a feature of the technology. It could be the extent to which the training could resemble or seem like the traditional fire training or a real fire.

As for the visual realism, it was mentioned that the graphics were good, but they could be better. This is a technological feature which will improve with the advancement of technology. The evaluation of the graphics could be related to the trainees’ previous experience with VR applications, particularly those who have experience with gaming and who may be used to high quality graphics. However, most trainees did not have prior experience with VR. Therefore, the topic of visual realism, specifically the graphics, was not raised as often as other elements such as fire, smoke and heat that are generally expected by everyone. In terms of audio realism, the VR training did have sound effects, but was not mentioned by any trainee which could either mean that they do not place importance on it or that the system managed to provide good audio realism. In terms of haptic realism, the subtheme of technical features showed that there were some features missing such as feeling the pressure in the hose or not having to pull the safety pin, as shown by the comments. Thermal haptic feedback was also missing, and this can influence realism (Nam et al., 2005; Barbosa et al., 2017). As for the interactive fidelity, the comments about missing the live reaction of the flame showed that they did not perceive the flame behavior in response to their actions to be realistic. Indeed, one of the characteristics of VR serious game is that it provides a dynamic and interactive training with instant cues and feedback (Oliva et al., 2019) and therefore, it is important to deliver this experience. The developers had studied the flame behavior and had incorporated that into the training in collaboration with research institutes. However, this comment could show that for those who possibly have experience with fire and safety trainings or those who have had prior real fire training, this may need further development. This is in line with the related work suggesting that the level of realism must be adjusted to user’s skill level (Garcia-Valle et al., 2017) and gaming experience (Walkowiak et al., 2015). It is possible that those who have more experience with technology may be better able to provide specific comments and notice that the visual graphics could be better, while those who have experience with real firefighting may notice the lack of pressure in the hose or certain technical issues with the equipment and flame reaction. These trainees are able to be more specific. However, there were comments that the training “seems real” without being specific; such trainees may not have prior specific experience and they subconsciously perceive realism because all the sensory modalities fulfilled the level of fidelity and realism required to replicate the real world. Therefore, experience and individual differences could play a role in how realism was perceived.

It is crucial to note that the most prevalent issue with realism was the lack of heat, smoke and flame behavior. This implies three points to consider. Firstly, it suggests that the technology still needs to mature in order to provide real time experience of heat, smoke and instant flame reaction. The programming of a myriad of real time flame reaction can be challenging. Providing smoke in a VR setting requires more graphical advancement since real smoke in a VR setting undermines its environmental friendliness. As for the heat, the developers mentioned that they are producing suits that can resemble the feeling of heat. Therefore, the technology is still maturing, and more features will be added to create multimodal fidelity and a sense of realism.

Secondly, with regard to the previous point, one needs to ask to what extent it is realistic to train on fire extinguishing in any setting, be it VR or the real fire training, where everything to the smallest detail is accounted for, including wearing full protective gear or picking up an already selected fire extinguisher capsule to fight that specific type of fire. It could be argued that none of the training methods described here are completely realistic in the sense of training on the specific situations that could occur at work. It could be that the different types of simulations (VR or traditional training) have different limitations and strengths. An advantage of VR training is that you are put in a scenario where you start to fight the fire right away while in the real fire training, you are fully protected with suits and helmets and have a large area in which to operate, which is not very realistic either. Furthermore, the real fire training is outdoors while most people work at indoor facilities and fires are more likely to occur indoors. It is important to evaluate whether wearing a suit that simulates heat in the VR training would enhance the sense of realism or would undermine the effectiveness of the training by creating discomfort or distraction and therefore compromise immersivity.

Thirdly, it is important to be aware of the trade-off between the complexity (size and photorealism, as well as the amount and quality of sensory information and details that are designed and incorporated into VR training) with the amount of interactivity and functionality (Wilson, 1997). With more built-in features, the frame rate update, temporal constraints, system lag, and consequently interactive fidelity might become slower, reducing the sense of realism. This may in turn reduce acceptance and eventually the use of the technology (Wilson, 1997). It will also become more expensive and time consuming to develop the VR training.

For these reasons, it is critical to determine at the design stage what would take priority and would enhance the effectiveness of the training, rather than providing the most realistic looking environment. This is a difficult balance to achieve because fidelity and immersion increases acceptance and perceived usability (Alexander et al., 2005) but, as Salas et al. (2012) suggest, the physical fidelity is not as essential as the psychological fidelity or a sense of presence fostered through the customized design of the simulation and scenarios, and the provision of instruction, measure of performance and timely feedback (Salas et al., 2012). Presence influences performance (Slater and Wilbur, 1995), which is the main goal for industrial training where interactive fidelity is essential. Perhaps heat, smoke and flame should be seen as elements of interactive fidelity rather than visual fidelity or olfactory fidelity. Therefore, designing effective training could greatly benefit from extensive task analysis and goal setting to see which elements must be salient to create the necessary level of realism and avoid redundancies that could compromise interactive and functional fidelity. It is important to consider the work by Chalmers et al. (2009) that suggested that only those sensory modalities that are more salient at that particular moment should be provided with high fidelity to reach multisensory realism. This indicates that this usability evaluation can help determine when is each sensory modality more salient.

### Emotional Experiences During the VR Training

Presence also depends on psychological and individual factors. Therefore, it is important to take the differences of emotional and bodily experiences into account when implementing trainings. The two subthemes of emotional experiences reflect on a range of emotions that were reported, from “excited” and “engaged” to feeling “ok” to stressed and “disoriented.” It also overlaps with the theme of bodily discomfort and simulator sickness. Emotional experiences of joy could be undermined by feelings of bodily discomfort, resulting in feelings of disorientation and stress. There is an overlap between these two themes, although they may have different underlying causes.

It is interesting to see how some trainees became more engaged and focused while others became less engaged and less focused. Adding heating jackets to provide thermal feedback or vibration, as well as other sensory feedback such as audio and olfactory feedback could enhance realism and engage the user in such a way that they take the game more seriously and less of a mere game (Rüppel and Schatz, 2011). This could also enhance emotional arousal in the VR serious game, leading to higher engagement (Chittaro and Buttussi, 2015) Furthermore, their prior expectation could play a role in the sense that their expected level of realism could influence their judgment of the VR training. Furthermore, perhaps trainees differ on what they focus on. Some could be more focused on the task while others focus on the VE and the surrounding. They were also provided with feedback on their performance by the VR system and the trainer, while being observed by other trainees. This could also influence their emotional experiences. In general, the pressure in the VR training was lower than the real training with real fire, wearing special suits, dealing with real flames, smoke and wind. This could be why most people reported feeling “Ok” or “good.”

### Bodily Experiences and Simulator Sickness During the VR Training

The experience of simulator sickness could overlap with feeling stressed and disoriented. Rebenitsch and Owen (2016) reported simulator sickness to be one of the most important human factors issues related to VEs; in their review, the reported likelihood of symptoms associated with VE ranges from 30 to 80%. In this training, 10% of the trainees reported simulator sickness symptoms. This lower percentage could be due to the walking interaction that reduces simulator sickness (Lee et al., 2017) and being able to control the VE objects (Chen et al., 2011) in comparison to other VR applications. However, it should not be overlooked that there is still a proportion of trainees that do experience sickness. For those working in safety critical industries, simulator sickness effects could be a serious issue, if they experience the side effects for some time after the training, for hours or even days (LaViola, 2000). If they use a VR setting at their job, it may be hazardous. Simulator sickness may limit the efficiency of the training (Rebenitsch and Owen, 2016) as it could discourage some users. Therefore, measures should be taken to reduce simulator sickness symptoms. This expands to technology, such as the reduction of system lag, in addition to individual factors. The latter is more difficult to address as there is no single agreed upon theory regarding why simulator sickness occurs. Nevertheless, organizations should consider whether preparatory training for learning how to stand, walk, act and interact with VE objects could improve trainees’ emotional and bodily experiences during VR training.

### VR Training as Supplementary Training

Although VR training is considered as good supplementary training to real fire extinguisher training, as it was not realistic enough there was a division on how effective it is and whether it could become an alternative in the future. However, we need to consider how realistic the traditional training is and how effective it would be in the long run. We need to consider whether we should replicate the traditional training or develop training geared toward skill improvement, stress management and memory enhancement to prepare for potential fires in the workplace. Ecological validity, which is the extent of similarity between trainees’ behavioral, cognitive and emotional response in simulated and real life training, does not necessarily require that trainees should believe that the simulated fire is real (Kinateder et al., 2014). Furthermore, not every sensory modality needs to be presented at the same fidelity level to reach realism (Chalmers et al., 2009). Therefore, if a satisfactory and effective training can be offered without replicating a traditional real fire training, then design of training can be altered and modified. This is a question for the beginning stages of training design to define its goal and direction and to communicate why they made such choices to trainees. This would prevent comparisons to traditional training.

### Implications for Further VR Developments for Training

It is vital for safety critical industries, management and end users to work closely with the designer and developers of VR training to form effective training methods. The first step would be to establish what makes the VR training acceptable to the end users. This study showed that realism through the incorporation of heat, smoke and flame is the most important theme. Perhaps the incorporation of these elements enhances both interactive fidelity and presence in effect, which is referred to by the trainees as realism. As a result, we must be aware of what kind of realism and what kind of effect we desire, without getting caught up in the terminology. Furthermore, administering a health check prior to training, regarding physical discomfort could better clarify the possible effect of the VR training on feeling discomfort and as part of a standard training procedure to avoid harm to those vulnerable to potential side effects such as simulator sickness. In addition to that, future research could aim to measure and compare the psychological responses in terms of stress and discomfort while engaging in real fire versus VR fire simulation, to see how stress levels are different in simulated fire versus real fire and how well the trainees can acclimate to this stress through VR. In order to have an effective training tool, it is also important to have a diverse group of end users from different backgrounds, experience with VR, gender and age. They can provide a wider range of feedback during the development process. It is more difficult than it seems to create a balance between available resources, functionality and effectiveness, total fidelity and realism. Close collaboration from the initial stage of task analysis and the continuous incorporation of human factors and feedback from the end users is essential. At the same time, sufficient investment and resource allocation is needed to design a training method that is effective, usable and that would save long-term costs. Finally, it is crucial to be aware of the possible effect of the group dynamics, training context and information dissipation about the training purpose and structure. Therefore, we would like to invite attention to not only the technical aspects of this method of training, but also the rhetoric that should be accompanying it.

### Future Research

Further research could test the time limit for VR training and VR exposure that would make the training effective and yet prevent simulator sickness. In addition to this, if there is a portion of the population that cannot even bear using VR, organizations must consider alternative modes of training without any consequences for the job performance and job security of these individuals. This is important to avoid any discriminatory practices caused by new modes of training. Future research could investigate the organizational measures that need to avoid discrimination when implementing new training methods using VR.

An expanded questionnaire and the possibility of conducting interviews with trainees could help the literature to understand the evaluations of VR training at the time of training, as well as after a period of time has passed. It would also be interesting to see how the evaluation of VR training could be different if the training started with a general VR orientation training session in which trainees learn how to move, act and perform when they are in the VR training.

Future research can be improved by adopting a mixed method approach to understand the general evaluation of the training with regard to improved realism. This can be done after including heated jackets for example and then measuring realism evaluation with and without jackets. Both open-ended questions, in combination with a rating scale could be used to assign a degree of satisfaction. Further research could evaluate trainees’ perception of VR training after traditional fire training to see how it differs from the current study. Psychological responses in VR training and traditional training can be investigated for their potential differences. We can evaluate administering different trainings in terms of enhanced realism to novices to teach fundamental techniques, versus highly realistic training to those with more experience in fire safety to train with more complicated scenarios, techniques and even communication and teamwork in emergency scenarios. Future research could also investigate the gender differences in using VR set up and especially fire extinguisher to investigate the suitable weight for fire extinguisher capsule. This can be used for ergonomic studies regarding safety gears and procedures in real workplaces. Future research could also include scenarios where the cause of fire needs to be identified, and the right fire extinguisher agent needs to be chosen, as opposed to already included in the scenario. If the trainee fails to put out fire, an evacuation and navigation training should be added as well. This can offer a more thorough training.

Furthermore, we need to understand more about the causes of simulator sickness, such as bodily position and navigation strategies and its implications for both individual performances and organizations. This understanding will help developers provide more effective and usable VR training solutions that could be adjusted and tailored to the trainees, training goal and task performance requirements.

### Limitation of the Study

One of the limitations in the study was the time pressure for the completion of the training sessions in one workday; this meant that the survey had to be short and there was no chance of conducting interviews with the trainees. We attempted to arrange follow up interviews but since most people work offshore in the oil and gas industries, the response rate was quite limited.

Another limitation was that we were not able to determine the training effectiveness on the job as it is not feasible to have real fires in safety critical industries to evaluate the effectiveness level. Additionally, we were not aware of the impact of prior experience with VR or familiarity with performing in the VR environment on task performance. It may be that feelings of disorientation could be related to not knowing how to move or act in VE. Another limitation was the low number of female participants that would make it difficult to investigate gender differences in evaluation of VR training.

## Conclusion

The aim of this paper was to investigate how the trainees from safety critical industries evaluated the use of VR technology for fire extinguisher training. The results showed that more than half of the trainees positively evaluated the VR training and reported having positive emotions during the training session. However, the majority preferred traditional training or were neutral. The main two themes that the trainees evaluated differently were the realism of the training and their emotional experiences during the VR session. While VR training at this stage was seen as a good supplement to the real training, its lack of realism was a major disadvantage, but the environmental friendliness, health and safety benefits and the efficiency and convenience of this training were evident advantages to real fire training.

It is important that all the stakeholders involved, including the developers, training organizations and end users of diverse backgrounds, closely collaborate during the design and development of the training to receive and accommodate more feedback with regard to what makes the training more effective with respect to the goal of training and the salient features needed to make it sufficiently realistic. Adequate resource allocation by organizations, the incorporation of human factors and continuous collaboration and improvement could elevate the VR training from a supplement to a potential alternative to real training in the future and enhance its acceptance by users and transference to the workplace in case of safety hazards.

## Data Availability Statement

The raw data supporting the conclusions of this article will be made available by the authors, without undue reservation.

## Ethics Statement

The studies involving human participants were reviewed and approved by Norwegian Centre for Research Data (NSD). The patients/participants provided their written informed consent to participate in this study.

## Author Contributions

MS was responsible planning and design of the study, data collection, analysis, and writing. KL was responsible for provision of funding, planning and the design of the study, and provided feedback on all phases including writing. RA was involved in data collection. MRS reviewed and provided feedback during the writing phase. All authors contributed to the article and approved the submitted version.

## Conflict of Interest

The authors declare that the research was conducted in the absence of any commercial or financial relationships that could be construed as a potential conflict of interest.

## References

[B1] Al-Adawi M. Luimula M. (2019). “Demo paper: virtual reality in fire safety-electric cabin fire simulation,” in *Proceedings of the 10th IEEE Int. Conf. Cogn. Infocommunications, CogInfoCom 2019 - Proc*, (Piscataway, NJ: IEEE), 551–552. 10.1109/CogInfoCom47531.2019.9089938

[B2] Alexander A. L. Brunyé T. Sidman J. Weil S. A. (2005). From gaming to training: a review of studies on fidelity, immersion, presence, and buy-in and their effects on transfer in pc-based simulations and games. *DARWARS Training Impact Group* 5 1–14. 10.5171/2012.800962

[B3] Andreatta P. B. Maslowski E. Petty S. Shim W. Marsh M. Hall T. (2010). Virtual reality triage training provides a viable solution for disaster-preparedness. *Acad. Emerg. Med.* 17 870–876. 10.1111/j.1553-2712.2010.00728.x 20670325

[B4] Bachen C. M. Hernández-Ramos P. Raphael C. Waldron A. (2016). How do presence, flow, and character identification affect players’ empathy and interest in learning from a serious computer game? *Comput. Hum. Behav.* 64 77–87. 10.1016/j.chb.2016.06.043

[B5] Backlund P. Engstrom H. Hammar C. Johannesson M. Lebram M. (2007a). “Sidh - a game based firefighter training simulation,” in *Proceedings of the 2007 11th International Conference Information Visualization (IV ’07)*, (Piscataway, NJ: IEEE), 899–907. 10.1109/IV.2007.100

[B6] Backlund P. Engström H. Johannesson M. Lebram M. (2007b). “Games and traffic safety - an experimental study in a game-based simulation environment,” in *Proceedings of the International Conference on Information Visualisation*, (Piscataway, NJ: IEEE), 908–914. 10.1109/IV.2007.54

[B7] Barbosa L. Monteiro P. Pinto M. Coelho H. Melo M. Bessa M. (2017). “Multisensory virtual environment for firefighter training simulation: study of the impact of haptic feedback on task execution,” in *proceedings of the EPCGI 2017 - 24th Encontro Portugues de Computacao Grafica e Interacao*, (Piscataway, NJ: Institute of Electrical and Electronics Engineers Inc), 1–7. 10.1109/EPCGI.2017.8124316

[B8] Biocca F. (1992). Will simulation sickness slow down the diffusion of virtual environment technology? *J. Presence-Teleop Virt.* 1 334–343. 10.1162/pres.1992.1.3.334 32495221

[B9] Blascovich J. Loomis J. Beall A. C. Swinth K. R. Hoyt C. L. Bailenson J. N. (2002). Immersive virtual environment technology as a methodological tool for social psychology. *Psychol. Inq.* 13 103–124. 10.1207/S15327965PLI1302_01

[B10] Bliss J. P. Tidwell P. D. Guest M. A. (1997). The effectiveness of virtual reality for administering spatial navigation training to firefighters. *Pres. Teleoperators Virtual Environ.* 6 73–86. 10.1162/pres.1997.6.1.73 32495221

[B11] Bode N. W. F. Kemloh Wagoum A. U. Codling E. A. (2014). Human responses to multiple sources of directional information in virtual crowd evacuations. *J. R. Soc. Interface* 11:91. 10.1098/rsif.2013.0904 24258157 PMC3869162

[B12] Bowman D. A. McMahan R. P. (2007). Virtual reality: how much immersion is enough? *J. Comput.* 40 36–43. 10.1109/MC.2007.257

[B13] Braun V. Clarke V. (2006). Using thematic analysis in psychology. *J.Qual. Res. Psychol.* 3 77–101. 10.1191/1478088706qp063oa 32100154

[B14] Brinck T. Bunyan J. Gergle D. Wood S. Blythe D. (2002). *Designing Web Sites that Work: Usability for the Web.* Available online at: https://books.google.com/books (accessed September 10, 2020).

[B15] Burdea G. C. Coiffet P. (2003). *Virtual Reality Technology*. New York: John Wiley & Sons.

[B16] Calp M. Enfiyeci O. Canal M. R. (2012). “Usability analysis of education-instruction institutions websites: a application study”. in *Proceedings of the 6th International Comput. Instructiom Technology Symposium*, (New York, NY: ACM).

[B17] Cao L. Lin J. Li N. (2019). A virtual reality based study of indoor fire evacuation after active or passive spatial exploration. *Comput. Human Behav.* 90 37–45. 10.1016/j.chb.2018.08.041

[B18] Cha M. Han S. Lee J. Choi B. (2012). A virtual reality based fire training simulator integrated with fire dynamics data. *Fire Saf. J.* 50 12–24. 10.1016/j.firesaf.2012.01.004

[B19] Chalmers A. Ferko A. (2008). “Levels of realism: from virtual reality to real virtuality,” in *Proceedings of the 24th Spring Conference on Computer Graphics*, (New York, NY: ACM), 19–25. 10.1145/1921264.1921272

[B20] Chalmers A. Debattista K. Ramic-Brkic B. (2009). Towards high-fidelity multi-sensory virtual environments. *Vis. Comput.* 25 1101–1108. 10.1007/s00371-009-0389-382

[B21] Chen Y. C. Dong X. Hagstrom J. Stoffregen T. A. (2011). “Control of a virtual ambulation influences body movement and motion sickness”. in *Proceedings of the BIO Web of Conferences.* (Les Ulis: EDP Sciences). 10.1051/bioconf/20110100016

[B22] Cheng K. Cairns P. A. (2005). “Behaviour, realism and immersion in games,” in *CHI’05 Extended Abstracts on Human Factors in Computing Systems*, (New York, NY: ACM), 1272–1275. 10.1145/1056808.1056894

[B23] Chittaro L. Buttussi F. (2015). Assessing knowledge retention of an immersive serious game vs. a traditional education method in aviation safety. *IEEE Trans. Vis. Comput. Graph.* 21 529–538. 10.1109/TVCG.2015.2391853 26357103

[B24] Chittaro L. Ranon R. (2009). “Serious games for training occupants of a building in personal fire safety skills,” in *Proceedings of the 2009 Conference in Games and Virtual Worlds for Serious Applications, VS-GAMES 2009*, (Piscataway, NJ: IEEE), 76–83. 10.1109/VS-GAMES.2009.8

[B25] Farra S. L. Miller E. T. Hodgson E. (2015). Virtual reality disaster training: translation to practice. *Nurse Educ. Pract.* 15 53–57. 10.1016/j.nepr.2013.08.017 24063793

[B26] Feng Z. González V. A. Amor R. Lovreglio R. Cabrera-Guerrero G. (2018). Immersive virtual reality serious games for evacuation training and research: a systematic literature review. *Comput. Educ.* 127 252–266. 10.1016/j.compedu.2018.09.002

[B27] Fernando Capretz L. (2014). Bringing the human factor to software engineering. *IEEE Softw.* 31 104–104. 10.1109/MS.2014.30

[B28] Frank L. H. Kennedy R. S. McCauley M. E. Root R. W. Kellogg R. S. (1984). *Simulator Sickness: Sensorimotor Disturbances Induced in Flight Simulators.* Orlando, FL: Naval Training Equipment Centre.

[B29] Gallace A. Ngo M. K. Sulaitis J. Spence C. (2011). “Multisensory presence in virtual reality: possibilities & limitations,” in *Multiple Sensorial Media Advances and Applications: New Developments in MulSeMedia (IGI Global)*, (Pennsylvania, PA: IGI Global), 1–38. 10.4018/978-1-60960-821-7.ch001

[B30] Gao Y. González V. A. Yiu T. W. (2017). “Serious games vs. traditional tools in construction safety training: a review,” Lean and Computing in Construction Congress (LC3): Volume I Ð Proceedings of the Joint Conference on Computing in Construction, (Greece: Heraklion Press) 653–660. 10.24928/jc3-2017/0070

[B31] Gao Y. Gonzalez V. A. Yiu T. W. (2019). The effectiveness of traditional tools and computer-aided technologies for health and safety training in the construction sector: a systematic review. *Comput. Educ.* 138 101–115. 10.1016/j.compedu.2019.05.003

[B32] Garcia-Valle G. Ferre M. Brenosa J. Vargas D. (2017). Evaluation of presence in virtual environments: haptic vest and user’s haptic skills. *IEEE Access* 6 7224–7233. 10.1109/ACCESS.2017.2782254

[B33] Gilbert S. B. (2016). Perceived realism of virtual environments depends on authenticity. *J. Presence-Teleop Virt.* 24 322–324. 10.1162/PRES_a_00276

[B34] Golding J. F. (2006). Motion sickness susceptibility. *J. Auton Neurosci.* 129 67–76. 10.1016/j.autneu.2006.07.019 16931173

[B35] Jiang L. Girotra R. Cutkosky M. R. Ullrich C. (2005). “Reducing error rates with low-cost haptic feedback in virtual reality-based training applications,” in *Proceedings - 1st Joint Eurohaptics Conference and Symposium on Haptic Interfaces for Virtual Environment and Teleoperator Systems; World Haptics Conference, WHC 2005*, (Piscataway, NJ: Institute of Electrical and Electronics Engineers Inc), 420–425. 10.1109/WHC.2005.111

[B36] Johnson D. M. (2005). *Introduction to and Review of Simulator Sickness Research.* Ansonia, CT: Farrel Corporation 10.1037/e456932006-001

[B37] Jorge V. A. M. Sarmiento W. J. Maciel A. Nedel L. Collazos C. A. Faria F. (2013). “Interacting with danger in an immersive environment: issues on cognitive load and risk perception,” in *Proceedings of the ACM Symposium on Virtual Reality Software and Technology, VRST*, (New York, NY: ACM), 83–92. 10.1145/2503713.2503725

[B38] Kinateder M. Ronchi E. Nilsson D. Kobes M. Müller M. Pauli P. (2014). “Virtual reality for fire evacuation research,” in *Proceedings of the 2014 Federated Conference on Computer Science and Information Systems, FedCSIS 2014*, (Piscataway, NJ: Institute of Electrical and Electronics Engineers Inc), 313–321. 10.15439/2014F94

[B39] Kobes M. Helsloot I. de Vries B. Post J. G. (2010). Building safety and human behaviour in fire: a literature review. *Fire Saf. J.* 45 1–11. 10.1016/j.firesaf.2009.08.005

[B40] Kraiger K. Ford J. K. Salas E. (1993). Integration of cognitive, skill-based, and affective theories of learning outcomes to new methods of training evaluation. *J. Appl Psychol.* 78 311–328. 10.1037/0021-9010.78.2.311

[B41] LaViola J. J. Jr. (2000). A discussion of cybersickness in virtual environments. *ACM Sigchi Bull.* 32 47–56. 10.1145/333329.333344

[B42] Lee J. Kim M. Kim J. (2017). A study on immersion and VR sickness in walking interaction for immersive virtual reality applications. *Symmetry* 9:78 10.3390/sym9050078

[B43] Lin J. W. Duh H. B. L. Parker D. E. Abi-Rached H. Furness T. A. (2002). “Effects of field of view on presence, enjoyment, memory, and simulator sickness in a virtual environment,” in *Proceedings IEEE Virtual Reality 2002*, (Piscataway, NJ: IEEE).

[B44] Lovreglio R. Duan X. Rahouti A. Phipps R. Nilsson D. (2020). Comparing the effectiveness of fire extinguisher virtual reality and video training. *Virtual Real.* 1:3 10.1007/s10055-020-00447-5

[B45] Månsson J. (2018). *Using a Virtual Fire Extinguisher as a Tool for Safety Training. Ph.D. Master Thesis.* Lund: Faculty of Engineering, Lund University.

[B46] Melo M. Bessa M. Roberto Frias R. (2016). “The impact of body position on the usability of multisensory virtual environments: case study of a virtual bicycle,” in *ACM International Conference Proceeding Series*, 20–24. 10.1145/3019943.3019947

[B47] Mól A. C. A. Jorge C. A. F. Couto P. M. (2008). Using a game engine for VR simulations in evacuation planning. *IEEE Comput. Graph. Appl.* 28 6–12. 10.1109/MCG.2008.61 18491708

[B48] Munafo J. Diedrick M. Stoffregen T. A. (2017). The virtual reality head-mounted display oculus rift induces motion sickness and is sexist in its effects. *J. Exp. Brain Res.* 235 889–901. 10.1007/s00221-016-4846-7 27915367

[B49] Nam C. S. Di J. Borsodi L. W. Mackay W. (2005). *A Haptic Thermal Interface?: Towards Effective Multimodal. Citeseer.* Available online at: http://citeseerx.ist.psu.edu/viewdoc/download?doi:10.1.1.465.8333&rep=rep1&type=pdf (accessed September 28, 2020)

[B50] Oliva D. Somerkoski B. Tarkkanen K. Lehto A. Luimula M. (2019). “Virtual reality as a communication tool for fire safety – experiences from the virpa project,” in *CEUR Workshop Proceedings*, (Paris: ISSN), 241–252.

[B51] Pausch R. Crea T. Conway M. (1992). A literature survey for virtual environments: military flight simulator visual systems and simulator sickness. *J. Presence* 1 344–363. 10.1162/pres.1992.1.3.344 32495221

[B52] Reason J. T. Brand J. J. (1975). *Motion Sickness.* London: Academic Press.

[B53] Rebenitsch L. Owen C. (2016). Review on cybersickness in applications and visual displays. *J. Virtual Real* 20 101–125. 10.1007/s10055-016-0285-9

[B54] Riva G. Davide F. IJsselsteijn W. A. (2003). “Being there: the experience of presence in mediated environments,” in *Studies in New Technologies and Practices in Communication. Being there: Concepts, Effects and Measurement of User Presence in Synthetic Environments (p. 5)*, eds Riva G. Davide F. IJsselsteign W. A. (Amsterdam: IOS Press).

[B55] Rüppel U. Schatz K. (2011). Designing a BIM-based serious game for fire safety evacuation simulations. *Adv. Eng. Informatics* 25 600–611. 10.1016/j.aei.2011.08.001

[B56] Salas E. Tannenbaum S. I. Kraiger K. Smith-Jentsch K. A. (2012). The science of training and development in organizations: what matters in practice. *J. Psychol. Sci. Public Interest.* 13 74–101. 10.1177/1529100612436661 26173283

[B57] Schuemie M. J. Abel B. van der Mast C. A. P. G. Krijn M. Emmelkamp P. M. G. (2005). *The Effect of Locomotion Technique on Presence, Fear and Usability in a Virtual Environment.* (France: EUROMEDIA).

[B58] Schwebel D. C. Gaines J. Severson J. (2008). Validation of virtual reality as a tool to understand and prevent child pedestrian injury. *Accid. Anal. Prev.* 40 1394–1400. 10.1016/j.aap.2008.03.005 18606271

[B59] Shaw E. Roper T. Nilsson T. Lawson G. Cobb S. V. G. Miller D. (2019). “The heat is on: Exploring user behaviour in a multisensory virtual environment for fire evacuation,” in *Proceedinghs of the Conference on Human Factors in Computing Systems - Proceedings*, (New York, NY: ACM). 10.1145/3290605.3300856

[B60] Sheridan T. B. (1992). Musings on telepresence and virtual presence. *J. Presence* 1 120–126. 10.1162/pres.1992.1.1.120 32495221

[B61] Sherman W. R. Craig A. B. (2002). *Understanding Virtual Reality: Interface, Application, and Design.* Cambridge, MA: Elsevier.

[B62] Slater M. (2003). A note on presence terminology. *Presence Connect* 3 1–5.

[B63] Slater M. Wilbur S. (1995). Through the looking glass world of presence: a framework for immersive virtual environments. *Five* 95 1–20.

[B64] Slater M. Khanna P. Mortensen J. Yu I. (2009). Visual realism enhances realistic response in an immersive virtual environment. *IEEE Comput. Graph. Appl.* 29 76–84. 10.1109/MCG.2009.55 19642617

[B65] Stanney K. Fidopiastis C. Foster L. (2020). Virtual reality is sexist: but it does not have to be. *J. Front. Robot. AI.* 7:4 10.3389/frobt.2020.00004 PMC7805626 33501173

[B66] Susi T. Johannesson M. (2007). *Serious Games-An Overview. Institutionen för Kommunikation och Information.* Available online at: www.americasarmy.com; (accessed September 15, 2020)

[B67] Tate D. L. Sibert L. King T. (1997). “Virtual environments for shipboard firefighting training,” in *Proceedings - Virtual Reality Annual International Symposium*, (Piscataway, NJ: IEEE), 61–68. 10.1109/vrais.1997.583045

[B68] Tichon J. Burgess-Limerick R. (2011). A review of virtual reality as a medium for safety related training in mining. *J. Health Safety Res. Practice* 3 33–40.

[B69] Uliano K. C. Kennedy R. S. Lambert E. Y. (1986). “Asynchronous Visual Delays and the Development of Simulator Sickness”. in *Proceedings of the Human Factors Society Annual Meeting.* Los Angeles, CA: SAGE, 10.1177/154193128603000502

[B70] Vicente K. (2003). *The Human Factor: Revolutionizing the way People Live with Technology.* New York, NY: Routledge.

[B71] Walkowiak S. Foulsham T. Eardley A. F. (2015). Individual differences and personality correlates of navigational performance in the virtual route learning task. *Comput. Hum. Behav.* 45 402–410. 10.1016/j.chb.2014.12.041

[B72] Weech S. Kenny S. Barnett-Cowan M. (2019). Presence and cybersickness in virtual reality are negatively related: a review. *J. Front. Psychol.* 10:158. 10.3389/fpsyg.2019.00158 30778320 PMC6369189

[B73] Wickens C. D. Lee J. Uu V. Becker S. G. (2004). *An Introduction to Human Factors Engineering* 2nd Edition (Washington, DC: Library of Congress Cataloging-in-Publication Data).

[B74] Wilcox-Netepczuk D. (2013). “Immersion and realism in video games-the confused moniker of video game engrossment,” in *Proceedings of CGAMES’2013 USA*, (Piscataway, NJ: IEEE), 92–95. 10.1109/CGames.2013.6632613

[B75] Wilson J. R. (1997). Virtual environments and ergonomics: needs and opportunities. *Ergonomics* 40 1057–1077. 10.1080/001401397187603

[B76] Witmer B. G. Singer M. J. (1998). Measuring presence in virtual environments: a presence questionnaire. *Presence* 7 225–240. 10.1162/105474698565686 32495221

[B77] Wouters P. van Nimwegen C. van Oostendorp H. van Der Spek E. D. (2013). A meta-analysis of the cognitive and motivational effects of serious games. *J. Educ. Psychol.* 105 249–265. 10.1037/a0031311

[B78] Xu Z. Lu X. Z. Guan H. Chen C. Ren A. Z. (2014). A virtual reality based fire training simulator with smoke hazard assessment capacity. *Adv. Eng. Softw.* 68 1–8. 10.1016/j.advengsoft.2013.10.004

[B79] Zou H. Li N. Cao L. (2017). Emotional response–based approach for assessing the sense of presence of subjects in virtual building evacuation studies. *J. Comput. Civ. Eng.* 31:04017028. 10.1061/(ASCE)CP.1943-5487.0000679 29515898

